# Beyond Coronativism: The Need For Agape

**DOI:** 10.1111/tesg.12438

**Published:** 2020-07-01

**Authors:** Annelies van Uden, Henk van Houtum

**Affiliations:** ^1^ Innovation Studies Copernicus Institute of Sustainable Development Utrecht University Vening Meineszgebouw A, Princetonlaan 8a 3584 CB Utrecht the Netherlands; ^2^ Department of Geography Nijmegen Centre for Border Research Radboud University P.O. Box 9108, 6500 HK Nijmegen the Netherlands

**Keywords:** Corona, b/ordering, inequality, autoimmunity, solidarity, agape

## Abstract

In this contribution we focus on togetherness, as one of the key notions in the current COVID‐19 crisis. Globally, it is seen as vital to stand and act together to combat the virus, and avoid a tragedy of the commons, in which actors are acting out of self‐interest and counterproductively to the general interest. In this essay we analyse the current geographical dissonant developments that the required human togetherness across the globe is facing. We find that the main conflicting tendencies, that we summarise as utilitarian locking up, nationalistic locking in and exclusionary locking out, are all employing a notion of togetherness which is largely based on an in‐group solidarity based on either age, gender, ethnicity, nationality or fitness. We argue that such narrow definition of togetherness falls short in dealing with the crisis in an effective as well as non‐discriminatory manner, and potentially could even lengthen or worsen the corona crisis. We end with a plea for a different conceptualisation of solidarity in the combat of the crisis, a radical non‐dividing form of togetherness: agape.

## Abstract 2

In this essay we explain how the aim to overcome the corona crisis through togetherness is impeded by three counterproductive bordering tendencies: utilitarian locking up, nationalistic locking in and exclusionary locking out. We argue that this ongoing geopolitical b/ordering and othering of people along the lines of age, gender, nationality or ethnicity, what we together coin coronativism, falls short in dealing with the crisis in an effective as well as non‐discriminatory manner, and could even have autoimmune effects in terms of global health as well as in terms of democracy and rule of law. To effectively combat the crisis, we make a plea for the employment of a different conceptualisation of solidarity, a radical non‐dividing form of togetherness,beyond nativistic divides: agape.

## Introduction – Together in Vulnerability

In the COVID‐19 crisis, also labelled as the corona crisis, one of the most often used words by government around the world to motivate the exceptional intervention strategies such as lockdown policies and the incremental unlock (or: exit) policies to deal with the crisis, is the word *together*. To minimise the number of casualties and in the meantime be able to win time for a vaccine to be developed, the United Nations and the World Health Organisation have plead for humankind all across the globe to work together, to stand as one, in order to protect the vulnerable (UN 2020; WHO 2020a, 2020b). And for this to work, solidarity of all of us would be required. In line with this, globally, we have seen the rise of national calls and even national legal enforcements of togetherness to maximally protect the elderly (children and young people are less affected in contrast to especially elderly men), and the vulnerable (especially heart, lung and diabetes patients), who face the highest risk of becoming seriously ill and to spread the pressure on the health care in the best possible way, such that every patient receives the care he/s/he needs radical measures were needed. And also to make sure that health care employees are not confronted with the horrific choice who to let live and who to let die, just because of capacity shortages.

In this paper we build on the burgeoning academic debate on the corona crisis by conceptually assessing the current societal challenges which the demanded policy of human togetherness is facing. In particular, we will build on and contribute to the literature related to the geopolitical b/ordering of solidarity and togetherness, that is, acting in opposition with and/or marginalising certain others (van Houtum & van Naerssen 2002; Vollan & Ostrom 2010; Sennett 2012; Jackson 2015; Bauder & Juffs 2020). To do this, we will be using a radical, humanistic approach (Burrell & Morgan 1979) with which we theoretically assess the socially constructed nature as well as the value bound axiology of the current politically used notions of solidarity. Using this radical, humanistic lens, in our conceptual exploration, we will critically discuss, illustrated with recent examples, the applied forms of togetherness in this crisis, ranging from a track and trace model to a complete lockdown (van Houtum & van Uden, 2020). After this, we will explore topical forms of counterproductive antagonisms that geographically, demographically and socially border the required togetherness. We will end with a short reflection on the autoimmune effects of this b/ordering of solidarity, what we, using a portmanteau, typify as *coronativism*, and argue that for a virus that travels beyond borders we also need a togetherness beyond socially constructed in‐groups – what we call ‘agape’, defined as love for mankind – if we wish to create a sustainable and just immunisation and exit strategy.

## The Tragedy of a Failing Solidarity in the Commons

Although it has not literally been framed as such, one could recognise in the urgent global call for ‘togetherness’ the classic philosophy of the ‘tragedy of the commons’ (Hardin 1968; Ostrom 1990). The concept of the ‘tragedy of the commons’ stems from the work done in human ecology initiated by Hardin (1968). Subsequently, this concept has also been applied to various fields and issues in social and economic research (see e.g. Ostrom 1990; Frischmann 2018; Frischmann *et al*. 2019). In general, the tragedy of the commons refers to problems that come about when a common‐pool resource, which can be accessed by a large number of people free of charge, is overused and this overuse destroys the resource itself. Examples relate to the harvesting of fish in public lakes and seas, greenhouse emissions in the global atmosphere and the use of public health care. Each individual makes his/her own decision about how much to use of this resource, that is, how many fish to catch or, when applied to this crisis, how much medical supplies and health care capacity to use. However, if none of the individuals restrain their use of the resource, the resources will soon collapse as it falls short on capacity. If one limits oneself, but fellow citizens do not, the resource will also be depleted, which creates in the short run an incentive to take your maximum share. Thus, the idea is that if everyone pursues his/her own self‐interest the ultimate outcome will be worse for everyone, as it will hollow out the commons and thereby harm the interests of the general public. This mechanism is typified as the tragedy of the commons or, emphasising the depletion of the commons, the tragedy of the failure of the commons or the tragedy of the unregulated commons (Jensen 2007).

The Nobel prize winner in economics Elinor Ostrom (2003, 2009) has famously suggested that key factors in order to avoid this tragedy of the commons, are democratic and fair rules governing the commons’ use, especially when there is such a perceptible threat of resource depletion without available substitutes as is the case with medical care and medication in the corona crisis. Hence, she argues, what is necessary is local, contextual rules with built‐in incentives for responsible use and punishments for overuse in the defined commonality. All members of the same local community that make use of the commons should strive to achieve the same goal, namely to protect the commons of selfish overuse. In order to attain this, targeted local governance is needed in the form of tacit norms and/or explicit rules, institutions and laws that constitutes and enforces the solidarity that is called for. Typically, the political appeal to solidarity in the locality will often be based then on a contingent mix of indigenous identity, imagined community, tacit rituals and skills or rational recognition of the reciprocity and mutuality needed to achieve the common goal (Sennett 2012; Iorio 2013; Jarvis 2019 Bauder & Juffs 2020). Yet, what is typical of this corona crisis – and different from the membership of a locally bordered commons that Ostrom (1990) dominantly based her writings on – is that all human beings are perhaps unwillingly yet unavoidably member of the same global commonality. If everyone would live his/her life as normal during this global outbreak, including economic and social cross‐border and interregional travel and interaction, the common medical care capacity would fall short very quickly making the impact on society at large, in terms of the number of ill patients and deaths, in the end beyond control. So, communities may develop their own governance rules and procedures and that may be effective, within that context and for the short run, but in the case of the corona pandemic these local communities are unavoidably geographically and chronically embedded in a global interdependence when trying to keep control over the virus. In this corona crisis, to quote Mazzucato (2020), ‘you are only as healthy as your neighbour’, be it your local or national neighbours.

Yet, this vital togetherness, plead for by the WHO and UN, is still rather far from reality (UN 2020; WHO 2020a). Despite the calls to stand and act together, different nations have chosen different and largely centripetally oriented pathways to protect the commons of the public health care system (Anderson *et al*. 2020). In the following, we will discuss the dominant variations in the immunisation and exit strategies chosen and assess their effects on togetherness.

## Variations of Togetherness

### Track and isolate

In most Asian countries and later in also a country like Israel, the focus from the start was not so much on standing together as an anonymous united one but more on the targeting and public disclosure of infected individual citizens (Cohen & Kupferschmidt 2020). Instead of the whole society joining in a lockdown to protect the vulnerable, individuals were tracked and traced and when sick, isolated. This resulted in a massive and public testing – for instance by constantly checking people’s temperature when entering a building or a public space and communicating this publicly – and the use of apps to trace the exact contacts and mobilities of people. Whenever someone would fail the temperature test then s/he would be locked up for several weeks in special corona camps. Combat‐wise the track and tracing seems very efficient and effective. And society does not have to bear the burden of being locked up as a whole. Yet, technically, in many countries the model quickly proved to be insufficient. The tracking and tracing could not keep up with the infection rate among the population. As a result, many of these countries eventually had to combine it with a lockdown after all (Cohen & Kupferschmidt 2020). What is more, it is an immunisation strategy that, potentially also has severe autoimmune consequences, referring to a situation in which the strategy to protect the society eventually forms a threat to that same society (Borradori 2003; van Houtum & Bueno Lacy 2020). For one, in order to let it function effectively it would require a far‐reaching, almost panopticon‐like invasion of privacy and freedom (Foucault 2009, 2010). A government that follows and controls people's private moves and publicly shares their temperature, risks transforming society into an Orwellian paranoid system. This not only rewards indifference and egoism in society to a worrying extent but also leads to a distrust in the anonymous, possibly affected fellow citizen. And this could result in a grim and punishing dichotomy of the to‐be‐avoided‐sick versus the non‐sick people, which would thus directly harm the idea of togetherness, the anonymous but solidary we.

### Herd immunity

In our home country, the Netherlands, like in the UK, the chosen immunisation strategy at first was to create a so‐called herd immunity, which entails that the fittest in society would gain immunity and thereby protect the vulnerable (Cohen & Kupferschmidt 2020). But when it became clear to the general public that this immunisation strategy would lead to the autoimmune consequence of a massive amount of victims and would take many years, and thus would not painlessly and quickly lead to a herd but literally to a hurt community, the Dutch as well the British government transitioned into a lockdown strategy for their citizens and the closing of the national borders for most foreigners (Cohen & Kupferschmidt 2020). The lockdown was not complete in the sense that all citizens who did not feel ill could still go out for essential shopping and recreational purposes as long as they would keep a distance of less than 1.5 metres (NL) or 2 metres (UK) to others and would not go around in groups of more than 2 (UK) or 3 persons (NL) (Cohen & Kupferschmidt 2020). In the Netherlands this strategy was framed as an ‘intelligent’ lockdown, although it was not made clear what a ‘stupid’ lockdown strategy would look like. Currently, as an unlock strategy the Netherlands has announced to introduce a track and trace model (including perhaps a corona‐app) with which people and their contacts can be tracked down, while supposedly guaranteeing maximum privacy (Rijksoverheid 2020). It remains to be seen whether this is indeed feasible at all.

### Together at home

Most other countries in Europe like Italy, France, Spain, Portugal, Serbia and Austria but also various other countries around the globe, like Australia and New Zealand, have immediately chosen for a far‐reaching lockdown (Cohen & Kupferschmidt 2020). In many cases by declaring a state of emergency, in which, in line with the works of Carl Schmitt and Agamben, the ‘normal’ rule of law is temporarily transcended by extra‐legal, exceptional sovereign decisions aimed to mitigate the emergency, all citizens were geographically bounded and borders were closed for in‐bound travel (Agamben 2005, 2020; Schmitt 2005). Fitting with such a martial law, to legitimise this complete confinement – in which citizens were prohibited to freely and independently move around, other than via specific certificates and only for essential purposes – the presidents and primeministers of these countries publicly declared a state of emergency in special TV broadcasts. To justify the exceptional state of affairs, President Macron for instance declared in his national TV‐speech: ‘Nous sommes en guerre’ (we are at war) (Cohen & Kupferschmidt 2020).

To shut down a society by a lockdown, be it framed as ‘intelligent’ or as a martial act, is, when it comes to immunisation perhaps less targeted, because sick people will only be revealed when they report it themselves. But from the perspective of solidarity and togetherness it seems a less discriminatory alternative. After, all, with a public lock‐down everyone participates for the greater good of the elderly and vulnerable. What is more, it quickly became clear that this strategy had a flywheel effect on togetherness itself. The threat of a constantly looming, dangerous viral infection that potentially could hit every body gave a powerful boost to new forms of local and global solidarity (Zizek 2020). All kinds of neighbourhood‐delivery‐services were introduced to do some shopping for the people who had to stay in home‐quarantine. Music was being played for the elderly to cheer up their day. Private balconies and windows were used as public platforms to sing or make music together, like in the case of Italy. And it resulted in applause for those who kept the commons going: the people working in the so‐called vital sectors. Of course, as with every (new) normality, not everyone obeyed the rules. Some people went on the streets in bigger groups than was allowed or even held secret parties, something that governments condemned and framed as a‐social. The message was clear: together should be really together. No exceptions. Men or woman, young or old, sick or not sick: #stayathome. And the ones who did not obey to these rules, could expect substantial fines.

## Togetherness and Its Antagonists

From the above, it becomes clear that the call for togetherness has globally been interpreted rather differently. So, globally there is *synchronicity*, COVID‐19 has hit every country this year, but there is a lack of geographical *compatibility*, the various action strategies are spatially rather heterogeneous. What is complicating the togetherness further is that geographical entities at various scales are even explicitly competing with each other for resources and thereby directly disrupt the global togetherness needed. In the following, we discuss the three main divisive, interrelated forces that we currently see unfolding: neoliberal *locking* *up*, coronationalistic *locking‐in*, and exclusionary *locking* *out*.

### Utilitarian locking up of the non‐fit

To begin with, it has become clear that the plea for the new normal of solidarity, cooperation and togetherness has run into direct confrontation with prevailing neoliberal calculative reasonings. In this vein, some utilitarian critics have argued, reasoning with the premise that society should aim to create the largest utility for the largest number of people, that the costs related to the standstill of the economy in the long run would be much higher than the benefits of collectively saving the elderly and the vulnerable in the short run (Luimstra 2020). And hence, they argue, it would economically be unwise to close down the entire society, because the ones who potentially are facing the health and lethal consequences the most would also be the ones who in the long run would not yield the biggest profits to society at large. Some have therefore even provocatively argued, much against the Kantian (and medical) logic in which every human being is morally equal and worth saving regardless of his/her characteristics and behaviour, that we do not have to go at such length saving these people, because many of them would be 'fat' and 'smokers' and it was therefore their own fault that they ended up at the intensive care unit. And hence they could perhaps best be isolated (locked up) (Luimstra 2020), so that the rest of 'us' could continue with our normal lives.

But also between cities and regions in the same nation state, we have seen manifestations of antagonistic neoliberal competition logics. If we, for instance, take a closer look at our own home country, the Netherlands, we have seen that hospitals located in the region that was hit first by corona (the North of Brabant) could not necessarily rely on the solidarity of other regions. Many hospitals elsewhere in the country were accused of unwillingness to take up patients of the almost full intensive care units in the hospitals in the North of Brabant (De Volkskrant 2020). To a large extent, this unwillingness could be interpreted as the material consequence of continuous waves of neoliberal privatisation and deregulation for decades (Brown 2015; Sparke 2016; Garnham 2017). The upshot of this continuous privatisation of the public sector is that even commons like hospitals are nowadays part of a neoliberal competition. It implies that collaboration between hospitals, even if it is better or cheaper for the patient, is not easily permitted, because it would violate free competition. And indeed, for hospitals in the Netherlands it became clear that collaboration between regional hospitals and even between different departments within the same hospital cooperation and solidarity had to be reinvented, so that patients and information would not be locked up within the boundaries of one hospital or region and to ensure that hospitals would take over patients of other regions and information and supplies were readily shared.

How dire the outcome of this neoliberal market thinking in health care can be, also became apparent in terms of the stock of pharmaceutical supplies (Emanuel *et al*. 2020; Harvey 2020). The corona crisis has brought to light how the economic logics in national health care have created a vulnerable *locked‐in* dependency on for‐profit, billion‐dollar pharmaceutical companies (Harvey 2020). Some of these pharmaceutical companies as well as traders in medical supplies persisted in prolonging their monopoly rents, and some even tried to take extra profit by asking extortionate prices, notwithstanding such a whopping life‐threatening crisis like this, impacting people all over the world (Buranyi 2020; Oxfam International 2020). For many critical scholars this comes as no surprise, as the damaging and even deadly consequences of a harsh neoliberal application in health care had been shown and warned for extensively in critical health care debates for a long time already (see e.g. Lemke 2001; Dardot & Laval 2013; Brown 2015; Sparke 2016).

So, one could argue that a strict utilitarian take on the corona crisis, as is argued for and/or practised by some, risks turning the required solidarity into a Hobbesian kind of togetherness, in which the calculative pursuit of one’s personal interests of one’s health, hospital or medical company prevails (Kapeller & Wolkenstein 2013). This potentially creates an atomised society in which competition rather than cooperation is seen as meritable, which potentially constitutes an unsolidary dichotomy of the fit versus the non‐fit, the healthy young versus the vulnerable elderly, fit women versus obese men or whatever category is hit harder by the virus, in which the non‐fit should be locked up in their (nursery) homes, such that the fit and healthy can live their life as ‘normal’, creating a neoliberal survival of the fittest and in fact literally so, with the virus as a deadly invisible hand.

### Nationalistic locking in

The second force that erodes the required togetherness that we discern here is the persistence of nationalism in this global corona crisis, the so‐called *coronationalism* (Colijn 2020; van Hecke 2020). The virus itself is blind for national borders, skin colour, power positions, descent or richness, and as such is a‐political, as it could affect everyone everywhere. But the political fight against it has clearly made the national differences more manifest and has increased the geographical inequalities even further. It has become painfully evident that even in an urgent and truly global crisis like this the alignment of national combat policies and global coordination in the distribution of medicins and supplies is seriously failing. Put differently, to a large extent, the societal lockdowns have led to nationalistic locked‐ins.

Sometimes, we have seen tendencies of solidarity beyond members that merely belong to the specific in‐group, for instance when the Chinese Jack Ma Foundation and the Alibaba Foundation donated half a million mouth masks and other protective equipment to the Netherlands (Ministry of Wealth, Health Care and Sports, 2020). Or when the European Union implemented a fund for research of which the results should be shared globally (European Commission, 2020a). But mostly, and especially among nation states, what we have seen is a global anarchistic fight, sometimes coincided with wild west scenes in the battle for medical supplies like mouth masks and respiration devices, in which even piracy was not shunned as shown by reports about deliveries that were stolen; including orders that were stopped at the border; and prices of supplies that increased by tenfold (The Guardian, 2020). In addition, countries like the United States have even further undermined the international order by threatening to terminate their support to the WHO (Kupferschmidt & Cohen, 2020).

Also in the EU, the union that was actually founded with the aim to create solidarity and to protect the rule of law and human rights beyond borders, the national oppositions have been magnified during the corona crisis (Bozorgmehr *et al*. 2020). Again, an emergency situation resulted in an internal clash. The EU has lately faced three major crises, a financial crisis, a so‐called refugee crisis and now a corona crisis, and all three crises have derailed into a crisis of solidarity among member states (Bozorgmehr *et al*. 2020). And each time, the EU commission members emphasised, without much effect, the importance of international collaboration and unity of policies. Also in this corona crisis, vice‐president Frans Timmermans as well as other commissioners warned, if we do not do this together, we should not be surprised if the EU harms itself even to the extent that it could fall apart (Timmermans 2020). Clearly, there have been some examples of cross‐border solidarity in the EU. For instance, Germany – showing a similar kind hospitality and humanitarian aid as during the so‐called refugee crisis – generously took over several patients of the Netherlands in their hospitals close to the Dutch border (Bosman 2020). But unfortunately, what is standing out is again international distrust and lack of solidarity. The same as in the case of the financial and refugee crises, the northern more affluent member states were not really willing to help the southern member states like Greece, Italy and Spain (Bozorgmehr *et al*. 2020). And with every new crisis, the support for the EU in both northern member states (‘they did it to themselves’) as well as the southern states (‘again we are being left on our own’) seems to be decreasing further.

Also the contrasts between east and west in Europe have further inflamed because of the corona crisis. In particular, in Hungary and Poland, this crisis, like in the case of the call for a European‐wide redistribution of refugees, has led to a further self‐distancing from the rest of the EU and has strengthened the autocratic seizure of the national rule of law (Kosmehl 2020).

For Agamben (2020), this latter misuse of the corona crisis, by undemocratically seizing more power in a state of emergency, is typically what could happen in a crisis, and is something that he has recently warned for in no mistaken terms. What is more, he regards the emergency measures even to be worse than the risk of the disease itself. In his words, the measures would be ‘frantic, irrational, and absolutely unwarranted’ (Agamben 2020). For him **‘**It is blatantly evident that these restrictions are disproportionate to the threat from what is, according to the NRC, a normal flu, not much different from those that affect us every year. […] We might say that once terrorism was exhausted as a justification for exceptional measures, the invention of an epidemic could offer the ideal pretext for broadening such measures beyond any limitation' (Agamben 2020). So, Agamben sees in this what he labels as ‘disproportionate response’ a ‘growing tendency to use the state of exception as a normal governing paradigm’. For him and various critical scholars like him, the autoimmune risk of a nationally declared state of emergency that is deemed necessary to protect the nation is that a democracy effectively becomes a technocracy run by non‐democratically accountable experts, that decisions are opportunistically taken that are not directly related to the emergency at hand and lastly, that the temporary situation is prolonged deliberately and unnecessarily which eventually could result into autocracy.

However, in sharp opposition to Agamben’s opinion, it has widely been established by now that COVID‐19 cannot and should not be equated with a regular flu as it much more contagious and deadly. To call the response ‘frantic’ and ‘disproportionate’ can hence hardly be called an expression of solidarity. But, what we do indeed see happening, and this would be in line with his expectations, is that countries such as Poland and Hungary but also China have already pushed the boundaries of martial law to an autocratic level (Harari 2020; Rachman 2020). Similarly, elsewhere, elections have been postponed opportunistically like in Bolivia and the campaigning of the opposition has been blocked like in Guinea (D’Urbino 2020). In addition, some governments have already suggested to ‘enrich’ the border apparatus in terms of so‐called ‘immunity passports’ and the discrimination of travellers from certain states (WHO 2020c). This would only strengthen the nativist discrimination and global mobility‐inequality that is already engrained in the visa border regime, where people are judged on the basis of their place of birth in the attempt to acquire the permission to travel (van Houtum & Bueno Lacy 2020). Furthermore, right‐wing populists like Trump and Orban have been playing a populistic blame game, claiming that the spread of COVID‐19, what Trump illustratively discriminatively labelled ‘the Chinese virus’ (@realDonaldTrump 2020) at first, would be the fault of the cosmopolitan elite, foreigners and imaginatively open borders (Chung & Li 2020; Devakumar *et al*. 2020; White 2020). And we will probably will be seeing more examples of what could be called ‘pandemic populism’ around the globe in the time to come (Pfeifer 2020). When it comes to the EU, one could state that the strategy to achieve togetherness paradoxically threatens to tear the EU further apart. The EU commission , by way of vice‐commissioner Timmermans, recently raised the question how many avoidable and self‐provoked internal crises the EU can actually survive. For him, the fear that the EU, by way of its own internal divisiveness, threatens to fatally harm itself, what recently has been called *Eucide*, becomes more and more real (van Houtum & Bueno Lacy 2020).

In short, what we see happening is that the required global togetherness is fiercely impeded by the national b/ordering of solidarity, a thinking in terms of ‘our nation first', as well as by all kinds of opportunistic misuses of the corona‐crisis by populist leaders, both of which have the potential to adversely exclude and marginalise people not belonging to the own imagined community (Anderson 1991; van Houtum & van Naerssen 2002; Featherstone 2012). This brings us to the third divisive force.

### Locked out: exclusive togetherness

To what degree the lack of geographical unity beyond local and national borders is amplifying the failure of the tragedy of the commons, will only be known when the crisis is over. What however is already becoming clear, is that some parts of the world and some population groups are clearly more vulnerable than others.

To wit, nations that are highly dependent on tourists are hit hard (McKenzie 2020). Especially, for the new tourist‐destinations that have emerged around the globe and moved out of poverty just because of their tourist‐industry, this means a serious setback (Gössling *et al*. 2020). But, perhaps even more poignant is that the adagium of #stayathome that was dominant and to some extent still is a guiding principle, at least until a cure is found, is a luxury that many people cannot afford (Bryant 2020; Meade 2020; Yancy 2020). The poorest on this planet have in fact been confronted with choosing between two evils: a lockdown without any opportunity to gain sufficient income and thus, in the worst case scenario, die of starvation; or the threat of being infected by the virus due to a lack of protection when moving around in the city to earn some money. An unbearable choice between life and livelihood. Also, inhabitants of slums are proportionally more vulnerable when exposed to the virus simply because they do not have a proper shelter to stay at home, running water to wash their hands or because there is no option to go into self‐quarantine when sick (Bryant 2020; Meade 2020; Wurcel *et al*. 2020). On top of that, in a lot of the less affluent parts of the world, but also in the richer countries like the US (Yancy 2020), access to health care and the extent to which the government can provide support is not up to par (Hopman *et al*. 2020; Deslatte 2020; Reyes *et al*. 2020; Thebault *et al*. 2020; Thomas and Anoruo 2020; Yancy 2020). Recent findings suggest that people with lower incomes, who are more often suffering from chronic health conditions such as obesity, diabetes or heart disease, are likelier to catch the disease, and are also likelier to die from it (WHO 2019; Fisher & Bubola 2020). As a result, it has already been established that the coronavirus is disproportionately affecting and killing African Americans (WHO 2019; Fisher & Bubola 2020) as they are more often living in poorer areas characterised by high housing density, high crime rates and poor access to healthy foods (Havranek *et al*. 2015; Yancy 2020).

And what to think of a possible corona outbreak in the refugee camps on the borders of the EU? The inhumane camps are the heartbreaking consequence of a lack of solidarity in the distribution of the reception of refugees, the previous crisis in the EU (Bozorgmehr *et al*. 2020; Kelly *et al*. 2020; van Houtum & Bueno Lacy 2020). The refugees are now yet again left to their own devices, with potential devastating lethal consequences (Bozorgmehr *et al*. 2020; Brandenberger *et al*. 2020; Hopman *et al*. 2020). This comes in sharp contrast to the rich and super rich whofled to their second or third homes, to private islands, private bunkers, or decided to stay for months on their superyachts (McKeever 2020). In other words, we have seen the rise of incredibly affluent corona refugees, that fled without all the deprivation, loss of freedom and hardship that most ordinary refugees suffer from. How the greatest utility for the greatest number of people in practice can come down to the greatest benefit for the smallest amount of people. Indeed, the virus potentially hits us all, but because of the (self)exclusionary and discriminating practices, clearly in a grossly unequal manner. It is telling that in most speeches held by presidents and prime‐ministers, these (international) vulnerabilities are not or only very scarcely given attention. It is not unlikely therefore that when a vaccine or medicine will become available, we will be seeing only a prolonging of the othering of the already vulnerable (van Houtum & van Naerssen, 2002; Mazzucato & Torreele 2020), with adverse effects on global inclusiveness. It is for the marginalised, the poor, the refugees, the homeless, and the (ethnically) discriminated or excluded that the call for international solidarity and the idea of togetherness in this corona crisis is tested the most (Bozorgmehr *et al*. 2020).

## Conclusion: The Need For Agapeic Togetherness

While the call of togetherness by governments is loud and widespread, it has become clear that its practical implementation is still obstructed by policies geared towards expanding merely an in‐group belonging, shared identity and reciprocity (Hunt & Benford 2004; Routledge *et al*. 2007; Iorio 2013; Rahbari 2019; Bauder & Juffs 2020). Such a solidarity, although internally potentially effective, runs the risk of creating or enlarging a bordered togetherness and exclusive reciprocity and hence a medical care only attainable for the ones that are part of that particular in‐group. The result of this prevailing identity‐oriented notion of togetherness is that it reduces and borders membership to those who belong to the own imagined community and exclude others who do not (van Houtum & van Naerssen 2002; Featherstone 2012). Yet, what the virus‐outbreak in a paradoxical way has shown is the interdependence of humanity beyond socially constructed divides. And this interdependence is both a threat as well as a necessary remedy. Because of our intrinsically globally connected world and our living closely together in dense countries and cities we are vulnerable to the rapid spread of viruses across the globe. A togetherness based on geographical, economic or ethnic divides, will therefore not help to solve the crisis, and could even have autoimmune effects in terms of democracy, rule of law and inequality. At the same time, it is also this very interdependent togetherness, beyond nativistic divides, beyond what we term *coronativism*, that should be seen as the most powerful and vital source we currently have to combat a virus like corona.

Reacting to Agamben’s (2020) warning for the ‘disproportionate response’ possibly leading to more autocratic control, Slavoj Zizek (2020) recently argued that ‘the measures necessitated by an epidemic should not be automatically reduced to the usual paradigm of surveillance and control’. Indeed, he argues, ‘quarantines and similar measures, of course, limit our freedom, and new Assanges are needed here to bring out their possible misuses’, but ‘such a social interpretation doesn’t make the reality of the threat disappear.’. For him, to only criticise the curtailing of one’s own freedom risks missing the paradox: ‘not to shake hands and to go into isolation when needed is today’s form of solidarity’. And so what we need, he argues, is an approach that ‘should reach well beyond the machinery of single governments: it should encompass the local mobilisation of people outside state control as well as strong and efficient international coordination and collaboration’.

We very much agree with this plea for a togetherness that goes beyond in‐group divides or local communities. The commons that the world is trying to protect from being affected by a globally spread virus, namely human life itself, cannot be exclusively related to any specific in‐group, but is unavoidably a vital concern of us all. It calls for a togetherness in which people realise that their ‘lives and actions affect one another in a web of institutions, interactions, and unintended consequences and acknowledge that they are together in a space of mutual effect’ (Young 2000, p.110). Put differently, a virus that is by its very nature politically blind calls for a combat strategy that should be equally blind for political divides.

In ancient Greece they had a beautiful term for such a non‐dividing togetherness: agape. It refers to one of the seven forms of love that were defined, next to for instance philia (friendship) and eros (romantic love) and stands for an overarching form of love, love for humankind. Not merely based on reciprocity, group belonging or any other personal characteristic, but an all‐encompassing love (Iorio 2013; Pope 2013; Hummels *et al*. 2019), based on concrete benevolent actions and equal regard (Jackson 2015). A radical form of love, and as radical etymologically stems from radix, roots, we need to deroot, de‐navitisticate the togetherness and the geographical in‐group solidarity, because it is exactly this radical non‐dividing form of togetherness, which is needed to deal with the crisis that potentially affects us all, everyone, and everywhere. A common threat asks for a common response. And a common responsibility to protect equality and human rights now and after the crisis, independent of age, ethnicity, and on where you are born or located.

Thus, such an agapeic, radical togetherness is necessary for two principal reasons. First, as a virological remedy, because everyone depends on the efforts of others, no matter who or where, to let the policies succeed. And second, togetherness is needed because it signifies what could be called a social remedy, to let society at large survive and to halt processes of egoism and atomisation, which jeopardise creating or magnifying the tragedy of the failure of the global commons.

In a world that has gained social and economic prosperity due to international interdependencies and exchange and in which globally spread viruses can and will occur, working together across socially constructed divides to fight these global viruses is not a romantic wishful thinking but an absolute necessity to further shape the international alignment. The persistence of a rivalling health care, antagonistic nationalism, and global inequality will only prolong the rates of transmission and mortality for everyone (Fisher & Bubola 2020). For the demanded togetherness, we thus might need to refine and enrich the current concept of solidarity, as this is, we ascertain, still predominated by a thinking in terms of solidarity for an imagined community, an ‘us’ notwithstanding ‘them’, a populist ‘our country first’ kind of thinking. We would argue that the concept of agape, the love of humanity, could be seen as a solidarity beyond borders, and action‐oriented complementation of the equal moral worth of individuals on which the UN (as well as the EU) declaration of human rights is founded. As such it could be the normative foundation for global regulations of togetherness and, consequently, an open and fair access to medical care and vaccines (Benkler 2006).

A radical inclusive togetherness that moves beyond in‐group based solidarity and incidental charity would be a hopeful and vital message for a world that finds itself in such a devastating crisis, and it would be instrumental as a moral guidance in the new normality of tomorrow. Yet, to let such an agapeic togetherness flourish, a lot of work needs to be done still, because what is continuously imminent, is the notion to fight the virus first, nationally as well as internationally and in a largely incompatible manner. We would consider these bordering and othering tactics some of which we described above as counterproductive for the world of tomorrow (Harari 2020; Rachman 2020). They represent a stubborn and clinging on at all cost to the ideas of a past that never existed. In the same vein, neoliberal thinking in the commons of health care, which has been has been producing calculative agents, reproducing inequalities and a hollowing‐out of the health care sector (Lemke 2001; Dardot & Laval 2013; Brown 2015; Sparke 2016), will not be conducive for an agapeic togetherness.

The interdependence that is becoming even more apparent during this corona crisis, is perhaps thus also a chance for a reimagination of the global interconnectedness of humanity, calling for systemic changes in the governance of our global world order. And perhaps it can learn from and contribute to the interesting discussions about alternatives such as degrowth, solidarity economics and social innovations to name a few that are already taking place (see for instance D’Alisa *et al*. 2015; van der Have & Rubalcaba 2016; Jarvis 2019). And hopefully, the recent agapeic expressions of support – like China and Cuba who sent medical supplies and doctors to Italy and South Africa respectively (BBC 2020; World Economic Forum 2020); the IMF that implemented an emergency fund for the less affluent countries to support their health care (IMF 2020); academics across the world collaborating on COVID‐19, vaccine and medicine research; and the European Union who, after a lot of pain and struggle installed a sort of support package for the EU as well as a global fund to combat the virus (European Commission 2020b) will have an illuminating and contagious impact for this to develop and grow further.

As long as we regard human life as something we need to care for together, and not pass on the burden to the individual – the sick versus the non‐sick, the young versus the old, and the protected versus the vulnerable, or to less‐affluent regions or nations – we will be able to retain and preserve the necessary sustainable togetherness that is needed to overcome this crisis. If not, the powerful idea that only together we will get control over corona might derail in an atomised and vulnerable world of together alone.
